# Supplementary material for: High Fecal Contamination and High Levels of Antibiotic-Resistant Enterobacteriaceae in Water Consumed in the City of Maputo, Mozambique
Source: Biology (Basel). 2021 Jun 20;10(6):558. doi: 10.3390/biology10060558 (PMC8235334; doi:10.3390/biology10060558)
Supplement: Supplementary file 1 [file biology-10-00558-s001.zip › biology-1241050-supplementary.pdf]

Supplementary material

Table S1. List and evaluation intervals of the antibiotics used, according to CLSI (2017) criteria

Type of antibiotic	Antibiotic	Inhibition halo diameter (mm)		
		Susceptible	Intermedium	Resistant
Beta - lactams	Amoxicillin (AMX) 10 mg	>17	14-16	≤13
	Amoxicillin/Clavulanic acid (AMC) 30:10 mg	>18	14-17	≤13
	Ceftazidime (CAZ) 30 mg	>21	18-20	≤17
	Imipenem (IPM) 10 mg	>23	20-22	≤19
	Cefpirome (CPO) 30 mg	>18	#	≤14
	Aztreonam (ATM) 30 mg	>21	18-20	≤17
	Cefoxitin (FOX) 30 mg	>17	15-17	≤14
	Ampicillin AMP 10 mg	>17	14-16	≤13
	Cefotaxime (CTX) 30 mg	>26	23-25	≤22
Non-beta-lactams	Chloramphenicol (CHL) 30 mg	>18	13-17	≤12
	Tetracycline (TET) 30 mg	>15	12-14	≤11
	Gentamicin (GEN) 10 mg	>15	13-14	≤12
	Trimethoprim/Sulfamethoxazole (SXT) 1:19 mg	>16	11-15	≤10
	Azithromycin (AZM) 15 mg	>13	#	≤12
	Ciprofloxacin (CIP) 5 mg	>21	16-20	≤15

The guidelines of CCLS [22] do not show the intermedium range for this antibiotic

Table S2. Antibiotic resistance profiles of 44 isolates of *Enterobacteriaceae*

**Type of antibiotic**	**Antibiotic***	***E. coli* (n = 28)**		***Klebsiella* spp. (n = 16)**	
		**Susceptible**	**Non-Susceptible**	**Susceptible**	**Non-Susceptible**
**beta-lactams**	AMX (10 µg)	18 (64.3%)	10 (35.7%)	7 (43.8)	9 (56.3)
	AMC (20/10 µg)	24 (85.7%)	4 (14.3%)	6 (37.5)	10 (62.5)
	CAZ (30 µg)	25 (89.3%)	3 (10.7%)	14 (87.5)	2 (12.5)
	IPM (10 µg)	17 (60.7%)	11 (39.3%)	7 (43.8)	9 (56.3)
	CPO (30µg)	28 (100%)	0	14 (87.5)	2 (12.5)
	ATM (30 µg)	27 (96.4%)	1 (3.6%)	12 (75)	4 (25)
	FOX (30 µg)	23 (81.1%)	5 (17.8%)	9 (56.3)	7 (43.8)
	AMP (10 µg)	15 (53.6%)	13 (46.4%)	8 (50)	8 (50)
	CTX (30 µg)	25 (89.3%)	3 (10.7%)	9 (56.3)	7 (43.8)
**Non-beta-lactams**	CHL (30 µg)	22 (78.6%)	6 (21.4%)	15 (93.8)	1 (6.3)
	TET (30 µg)	14 (50%)	14 (50%)	6 (37.5)	10 (62.5)
	GEN (10 µg)	25 (89.3%)	3 (10.7%)	12 (75)	4 (25)
	SXT (23,75/1,25µg)	17 (60.7%)	11 (39.3%)	13 (81.3)	3 (18.8)
	AZM (15 µg)	24 (85.7%)	4 (14.3%)	11 (68.8)	5 (31.3)
	CIP (5 µg)	28 (100%)	0	14 (87.5)	2 (12.5)

*Amoxicillin (AMX) 10 mg; amoxicillin/clavulanic acid (AMC) 30:10 mg; ceftazidime (CAZ) 30 mg; imipenem (IPM) 10 mg; cefpirome (CPO) 30 mg; aztreonam (ATM) 30 mg; cefoxitin (FOX) 30 mg; ampicillin (AMP) 10 mg; cefotaxime (CTX) 30 mg; chloramphenicol (CHL) 30 mg; tetracycline (TET) 30 mg, gentamycin (GEN) 10 mg, trimethoprim / sulfamethoxazole (SXT) 1:19 mg, azithromycin (AZM) 15 mg; ciprofloxacin (CIP) 5 mg.
